# Novel use of balloon-tipped bronchial blockers to occlude neonatal tracheoesophageal fistula: a case series

**DOI:** 10.1186/s12887-022-03131-6

**Published:** 2022-01-25

**Authors:** Bo Sheng, Lin Zhong, Bin Du

**Affiliations:** 1 Department of Anesthesiology, West China Second University Hospital, Key Laboratory of Birth Defects and Related Diseases of Women and Children (Sichuan University), Sichuan University, Ministry of Education, City of Chengdu, 610041 Sichuan, China; 2 Department of Pediatric Pulmonary and Immunology, West China Second University Hospital, City of Chengdu, Sichuan University, 610041 Sichuan, China; 3 Department of Anesthesiology, West China University Hospital, City of Chengdu, Sichuan University, 610041 Sichuan, China

**Keywords:** Tracheoesophageal fistula (TEF), Balloon-tipped bronchial blocker (BTBB), Fogarty catheter, Fiberoptic bronchoscope, Case report

## Abstract

**Background:**

Management of the airway and ventilation in neonates with a tracheoesophageal fistula (TEF) remains a significant challenge. The routine method of intubation involves placement of the tracheal tube tip beyond the fistula opening followed by isolation of the fistula from ventilation using the inflated cuff. When the fistula opening is close to the carina or below the level of the carina, the traditional technique is not suitable for adequate ventilation. Moreover, this method fails to prevent gastric insufflation.

**Case presentation:**

We herein report a series of 10 newborns with TEFs (1,090–3,080 g) who underwent bronchoscopic insertion of a 5-Fr balloon-tipped bronchial blocker (BTBB) for temporary occlusion of the fistula. In seven newborns, placement of the BTBB was easily and quickly achieved with no incorrect placements. In addition, we successfully utilized the inner hollow cavity of the BTBB for gastric decompression in six neonates with severe gastric distension. However, three failed placements occurred in premature infants (<2,000 g) because the narrow cricoid cavity was too small to accommodate a 2.8-mm fiberoptic bronchoscope and a BTBB. The procedure was well tolerated by all infants, and no significant adverse events occurred.

**Conclusions:**

Our findings illustrate that BTBBs can provide durable blockage of the fistula opening and should be considered as a treatment modality for infants with large carinal TEFs. Moreover, BTBB placement is neither arduous nor time-consuming. The hollow center, small round balloon, and 30-degree angled tip of the BTBB make this device feasible for clinical application, especially for neonates with severe gastrointestinal distension.

**Supplementary Information:**

The online version contains supplementary material available at 10.1186/s12887-022-03131-6.

## Background

Esophageal atresia/tracheoesophageal fistula (EA/TEF) is one of the most common congenital malformations requiring surgical correction during the neonatal period. The high morbidity and mortality rates in these infants are always accompanied by aspiration, gastric distension, and ineffective ventilation. The anesthetic management of infants with TEFs focuses mainly on ventilation of the lungs while avoiding ventilation through the fistula. The traditional intubation technique involves placement of the tracheal tube tip beyond the fistula opening, isolating the fistula from ventilation using the inflated cuff [1, 2]. In a study of bronchoscopy in 113 patients with TEFs, Holzki [3] reported that the fistula was below the level of the carina or within 1 cm of the carina in 33% of patients. Under these circumstances, the routine intubation method is not suitable for adequate ventilation. Additionally, it fails to prevent gastric insufflation.

The Fogarty balloon catheter, a commonly used device, can provide effective ventilation supported by occlusion of the fistula in neonates with a large carinal TEF [4, 5]. In current anesthetic practice, the straight tip and solid center of the Fogarty catheter have limited its widespread used in infants with TEFs. In one study, the mean time for visual confirmation of correct Fogarty catheter placement was 4.48 min by rigid bronchoscopy [10], however, insertion of a Fogarty catheter using a fiberoptic bronchoscope (FOB) was found to be more laborious because of poor control of the straight tip and uncontrollable ventilation. Moreover, the solid center of the Fogarty catheter made it inconvenient to suction secretions and expel the gas from the lungs or the stomach. Alternatively, in another study, a pediatric bronchial blocker was safely used to isolate the fistula from ventilation in older children [6]. However, whether bronchial blocker technology is feasible for newborns has not yet been fully explored. We herein present our experience using a 5-Fr balloon-tipped bronchial blocker (BTBB) for temporary mechanical obliteration of TEFs in neonates. We propose this technique as a simple and promising method of intubation for infants with large, low-lying TEFs.

## Case presentation

After obtaining approval from the ethics committee of West China Second University Hospital (Approval No. 20200022gc), we collected the data of 10 neonates with TEFs who were treated in our hospital from 1 to 2019 to 31 September 2020. In all neonates, a 5-Fr BTBB (FA03E015110; Well Lead Medical, Guangzhou, China) was placed under general anesthesia using a fiberoptic bronchoscope (FOB) (BF-XP60 2.8 mm; Olympus, Tokyo, Japan), and occlusion of the fistula was performed under direct vision. The parents of all infants had consent-for-research authorization on file and gave their written consent for publication.

**Fig. 1:**
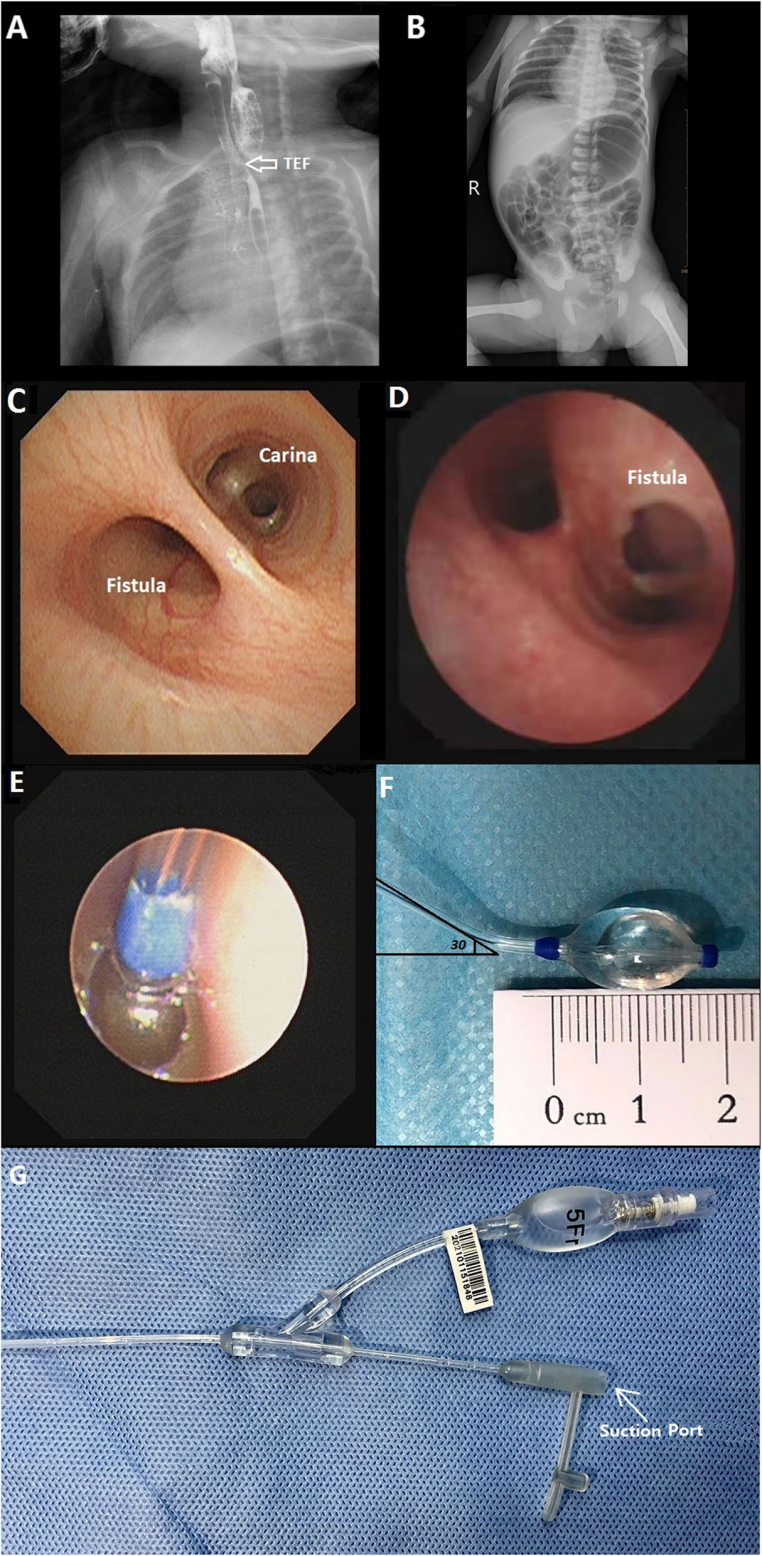
**A** Preoperative upper gastrointestinal contrast imaging. The white arrow indicates the opening between the esophagus and the trachea. **B** Posteroanterior chest radiograph shows severe gastrointestinal distension and bilateral pneumonia. **C** Bronchoscopic view of a TEF. The fistula opening is located 1 cm above the carina. **D** The opening of the fistula is located at the carina. **E** Under bronchoscopy, the balloon of the BTBB is inflated with normal saline, and the blue marker is clearly shown. **F** The size and angle of the 5-Fr BTBB are shown. **G** The suction port and pilot balloon of a 5-Fr BTBB are shown. TEF = tracheoesophageal fistula; BTBB = balloon-tipped bronchial blocker

Ten neonates were sequentially studied, and their detailed information is summarized in Table 1. Their median gestational age was 36 weeks (range, 29–40 weeks), and their age and weight at presentation ranged from 3 to 22 days and from 1,090 to 3,080 g, respectively. Among the 10 newborns, 5 (50%) were diagnosed with EA/TEF by prenatal evaluation and 6 (60%) had severe gastrointestinal distension. In addition to their history of cyanotic episodes, choking during feeding, coughing, gastrointestinal distension, and shortness of breath after birth, the preoperative diagnosis of TEF was supplemented by contrast radiography (Fig. 1 A). A posteroanterior chest X-ray was helpful for the diagnosis of gastrointestinal distension and aspiration pneumonitis (Fig. 1B).

**Table 1:** Clinical Characteristics of Patients

Case	Sex	Age (day)	Delivery (week)	Weight (g)	Gastrointestinal distension	Successful placement of BTBB	BTBB Size	Number of attempts	BTBB placement Time (min)	Gross Classification	ET Size (ID)	ET Depth (cm)	Carina-Fistula Distance (cm)
1	M	5	36	2520	Severe	Yes	5-Fr	1	3.18	C	3.0	9	1
2	M	10	39	2510	Severe	Yes	5-Fr	1	2.50	C	3.0	10	1
3	M	11	32	1940	Severe	No	5-Fr	1	4.48	C	3.0	8	1.5
4	M	3	40	3080	Slight	Yes	5-Fr	1	2.26	C	3.0	9	1
5	M	11	29	1090	Severe	No	5-Fr	2	5.36	E	2.5	8	0*
6	M	18	40	3000	Slight	Yes	5-Fr	1	2.31	C	3.0	10	1
7	M	3	36	2600	Slight	Yes	5-Fr	1	3.06	C	3.0	10	0*
8	M	7	38	2290	Slight	Yes	5-Fr	1	2.42	C	3.0	8.5	0*
9	F	7	36	2020	Severe	Yes	5-Fr	1	3.23	C	3.0	10	1
10	M	22	32	1560	Severe	No	5-Fr	1	4.12	E	3.0	9	2

*BTBB *Balloon-tipped Bronchial Blocker, *ET *Endotracheal Tube, 0*: The fistula is located at the carina

The procedure was performed in the operation room, and 0.04 mg/kg of atropine was given intravenously prior to the induction of anesthesia. After all necessary monitoring equipment (electrocardiograph monitor, pulse oximeter, noninvasive blood pressure monitor, and temperature probe) had been placed on the infants, fentanyl (1 mcg/kg) and propofol (1 mg/kg) were given by an intravenous injection, and anesthesia was induced by mask inhalation of 1–4% sevoflurane in 100% oxygen. Spontaneous respiration was maintained to the greatest extent possible. If apnea occurred, we immediately ventilated the infant with the face mask and avoided positive-pressure ventilation exceeding 10 to 15 cmH_2_O. After spraying the larynx and trachea with 1% lidocaine, we inserted a 1.0^#^ laryngeal mask airway, and a swivel connector was used to facilitate examination using the FOB.

Under direct bronchoscopic visualization, we found that most of the fistulas were located at the posterior tracheal wall or at the level of the carina, and the distance between the carina and fistula was 0 to 2 cm (Fig. 1 C and D). The 5-Fr BTBB was then cautiously inserted into the TEF tract through the fistula opening, and the balloon was inflated with saline solution for temporary mechanical obliteration of the fistula (Fig. 1E and F). A cuffed endotracheal tube (ET) with an inner diameter of 2.5 or 3.0 mm was inserted as the final step, and the depth of the ET was 8 to 10 cm from the central incisors. The ET and BTBB were firmly fixed. The end of the BTBB was divided into two separate components, a suction port and a pilot balloon (Fig. 1G). The suction port, connected to a hole in the BTBB tip, was used for gastrointestinal decompression in the neonates with severe gastric distension and pneumonia. After careful auscultation of both lungs and the stomach, 0.1 mg/kg of cisatracurium was administered intravenously, and the neonates were supported by a pressure-controlled ventilator (Datex-Ohmeda CS2; GE Healthcare, Chicago, IL, USA).

The EA/TEF was repaired by thoracoscopy or open thoracotomy without difficulty, and the procedure was well tolerated by all infants. When the surgeons prepared to ligate the fistula, the balloon of the BTBB was deflated and withdrawn from the TEF tract. All newborns remained intubated postoperatively and were transferred to the neonatal intensive care unit. Subsequent recovery was uneventful, and no significant adverse events occurred.

## Discussion and conclusions

A congenital TEF is a challenging, life-threatening condition, and the morbidity and mortality rates are much higher for complicated TEFs [1]. Surgical correction with closure of the communication between the trachea and esophagus is curative, but management of the intraoperative airway and ventilation are challenging for even the most experienced anesthesiologists [2, 7]. Preoperative bronchoscopy may help the anesthesiologist and surgeon to assess the size and location of the TEF and plan for the best airway management strategy [3]. In our case series, we inserted a laryngeal mask airway to facilitate examination by the FOB and assess the location of the fistula before intubation. Most of the fistulas were found at the posterior tracheal wall or at the level of the carina, and the distance between the carina and fistula was very short. Additionally, the routine intubation technique was not appropriate for ventilation of both lungs and gastric decompression; therefore, we chose a 5-Fr BTBB for mechanical obliteration of the fistula. Under direct visualization with a bronchoscope, we successfully placed the BTBB for obliteration of the fistula in 7 of 10 neonates. The mean time for placement of BTBB was 2.70 ±0.39 min in seven successful cases. Skill in its use was quickly acquired, and insertion became increasingly more rapid. The procedure was well tolerated by the infants, and no significant adverse events occurred.

Unfortunately, placement of the BTBB failed in three preterm infants (Cases 3, 5, and 10), most likely because the narrow cricoid cavity did not have enough space to accommodate a 2.8-mm fiberoptic bronchoscope and a BTBB. The cricoid is anatomically the narrowest part of the airway in preterm infants. An experimental study demonstrated that in premature infants with a gestational age of <32 weeks and body weight of <1,770 g, the diameter of the cricoid lumen was about 3.0 to 3.5 mm [8]. Although the laryngeal structures of preterm infants exhibit high elasticity, allowing the passage of a larger-diameter ET, we found that there was not enough space to accommodate a 2.8-mm FOB and a BTBB. We believe that the use of a smaller-diameter FOB (1.9 or 2.2 mm) might improve the success rate of BTBB placement in premature infants.

After placement of the BTBB failed in Cases 3 and 10, we inserted a cuffed endotracheal tube (ET) with an inner diameter of 3.0 mm into the right main bronchus in the traditional way, then gradually withdrew the ET until breath sounds were heard in both lungs and no gurgling noises were detected by auscultation of the gastric area. Mechanical ventilation was then started. To prevent inadvertent gastric distention during the operation, we used small rolled gauzes and bandages to compress the infant’s stomach instead of invasive gastrostomy decompression. Recent studies indicate that emergency gastrostomy decompression can exacerbate intraoperative hypoventilation in infants with TEFs because most of the air enters the gastrostomy site [9]. When placement of the BTBB failed twice in Case 5 (an extremely low-birth-weight premature infant), we inserted an uncuffed ET with an inner diameter of 2.5 mm into the right main bronchus and then gradually withdrew the ET. However, gurgling noises were continuously detected by gastric auscultation, and gastric distention became gradually aggravated during mechanical ventilation. Thus, we first used bandages to compress the infant’s stomach, and the accurate position of compression was determined by gastric auscultation where the gurgling noise was the strongest, or by ultrasound. To avoid excessive abdominal compression and decreased visceral perfusion, we used the pulse oximeter waveform of toes to determine whether the degree of compression was appropriate. Moreover, recent clinical practices have demonstrated that high-frequency oscillatory ventilation (HFOV) can be used intraoperatively to provide a still operation field with good oxygenation and CO_2_ elimination in TEF operation [2]. We then utilized HFOV at a frequency of 11 Hz (1 Hz = 60 times per min) to improve the ventilation and reduce the gastric distension. The whole procedure was uneventful, and subsequent recovery was good.

Although the Fogarty catheter has been a cornerstone in the management of complicated airways in infants with TEFs, there are some limitations when it is used as a conduit for occlusion of the fistula [5]. First, Fogarty catheters are expensive and solid, whereas BTBBs are more economical and have a hollow center to suction secretions and expel gas from the lungs or stomach. For newborns with severe abdominal distension, we found that BTBBs are more suitable than Fogarty catheters for relieving gastrointestinal distension. In current anesthetic practice, visual confirmation of correct placement of a Fogarty catheter using an FOB is a laborious process because of the straight tip [11]; however, coordination with a rigid bronchoscope allows direct visualization and good control of Fogarty catheter insertion. In contrast, the tip of BTBBs is designed at a 30-degree angle, and the direction to the target can be conveniently adjusted by twisting the catheter shaft; additionally, the blue marker at the tip facilitates easy identification using an FOB. Furthermore, recent studies have indicated that the balloon of BTBBs produces a lower “cuff-to-tracheal” pressure than the Fogarty catheter, decreasing the risk of esophageal necrosis and airway rupture [6, 10]. Another potential problem associated with the Fogarty catheter is inadvertent malpositioning into the trachea, which may contribute to ventilation failure or hypoxemia [11]. In contrast, dislodgement of the BTBB into the trachea was a rare phenomenon in our case series.

The use of BTBBs to enable single-lung ventilation has been described in pediatric thoracic surgery [6], but there are few data on its use in obliteration of TEFs. In the present study, a 5-Fr BTBB was a safe and promising method for infants with a large, low-lying TEF. The technique was successful for temporary obliteration of the TEF in neonates requiring surgical correction, and as experience is gained with more infants, it may offer a good solution for unstable neonates. The hollow center, small round balloon, and 30-degree angled tip make the BTBB feasible for clinical application.

## Supplementary Information

**Additional file 1**

## Data Availability

All data supporting the findings of this article are included within the article.

## Abbreviations

EA: esophageal atresia
TEF: tracheoesophageal fistula
BTBB: balloon-tipped bronchial blocker
ET: endotracheal tube
CO_2_: carbon dioxide
FOB: fiberoptic bronchoscope
HFOV: high-frequency oscillatory ventilation
